# The Trypanosome Nuclear Pore Reveals 1.5 Billion Years of Similarities and Differences

**DOI:** 10.1371/journal.pbio.1002366

**Published:** 2016-02-18

**Authors:** Richard Robinson

**Affiliations:** Freelance Science Writer, Sherborn, Massachusetts, United States of America

## Abstract

Comparison of the nuclear pore complex from trypanosomes with those of animals, plants, and fungi offers new insights into this quintessential eukaryotic structure. Read the accompanying Research Article.

The nuclear pore complex (NPC) is perhaps the most magnificent protein complex in the eukaryotic cell. It is built from almost 500 individual protein molecules of about 30 different types, arranged in 8-fold symmetry to create a central pore through which proteins, RNAs, and all other molecules must pass in order to enter or exit the nucleus. The nucleus and the nuclear membrane may define what it means to be a eukaryote, but it is the NPC that makes eukaryotic existence possible.

Given its functional importance, you might think that the NPCs of all eukaryotes would be the same—that the constraints of natural selection might have prevented any variation from some successful early model. But in this issue of *PLOS Biology*, Samson Obado, Mark Field, Brian Chait, Michael Rout, and colleagues show that the NPC of the parasite *Trypanosoma brucei* differs in some important ways from those of vertebrates, plants, and yeast (Fig 1). Their findings shed light on the evolution of the NPC, strengthening the argument that it may have originated as a far simpler progenitor.

**Fig 1:**
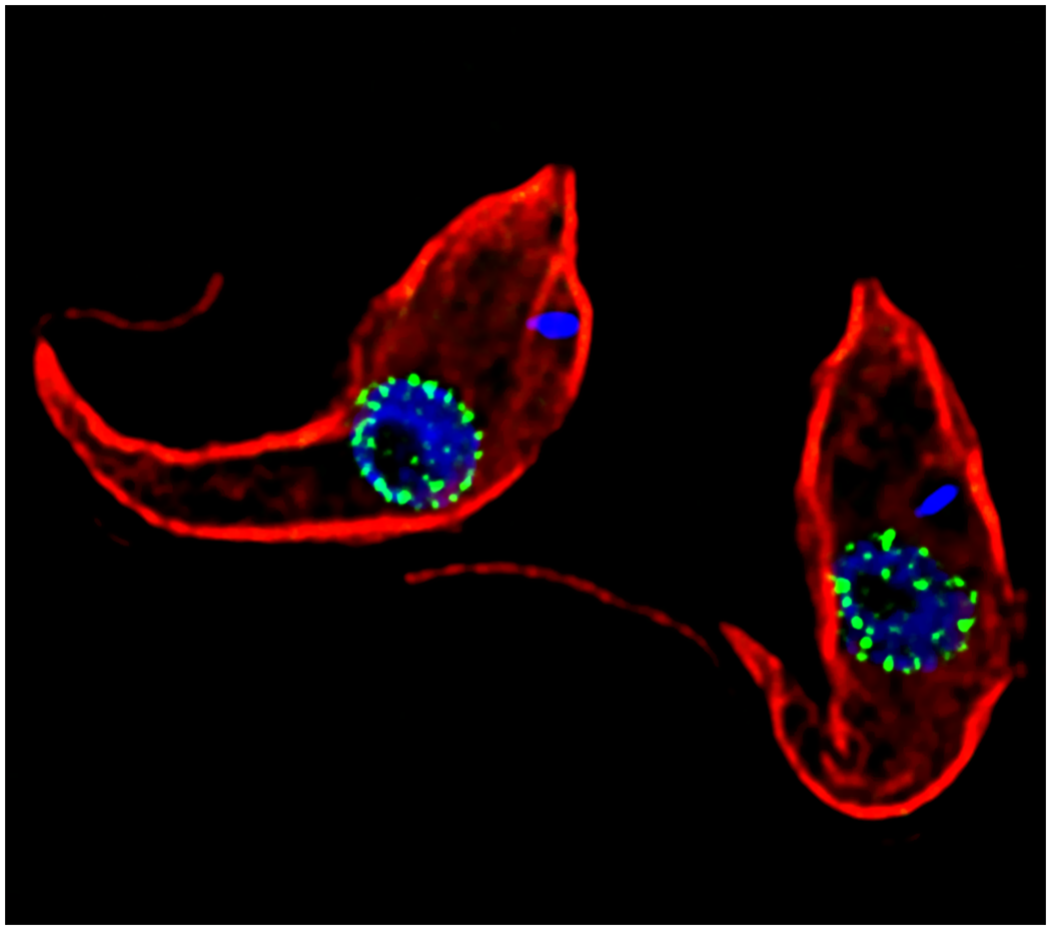
Conservation and divergence at the nuclear rim. The trypanosome surface is stained in red, with the nuclear pore complex highlighted in green and DNA highlighted in blue. Detailed proteomics dissection demonstrates a simpler form for the nuclear pore in trypanosomes and divergent function. *Image credit*: *Dr*. *Jeffery deGrasse*.

The choice to study *T*. *brucei*, which causes African sleeping sickness, was no random one—trypanosomes branched from the rest of the eukaryote lineage quite early. Vertebrates, plants, and yeast—whose NPCs have been characterized in detail—are more closely related to each other than any of them are to the trypanosomes. Thus, the new study provided an opportunity to compare the structural similarities and differences between groups that have been separated for over 1.5 billion years.

The authors’ group had previously identified 22 *T*. *brucei* nucleoporins (or Nups, as all the proteins of the nuclear pore complex are known), which they identified as orthologs (related by descent and presumed function) of Nups from yeast or humans. Here, they performed “affinity capture/mass spectrometry interactomics” to better characterize the functional interactions of the *T*. *brucei* Nups and to discover new ones.

The process of affinity capture uses a known protein as the bait to find the set of proteins to which it binds. Each Nup was tagged in succession with green fluorescent protein (GFP) (which, remarkably, did not interfere with the function of the pore complex) and was then purified and captured by an antibody to the GFP. Depending on the stringency of the purification process, the GFP-tagged Nup brought along with it one, some, or all of the other proteins to which it was bound, either directly or indirectly, in the various subunits of the NPC. By repeating this process with each known Nup, the authors were able to, as they put it, “walk out” from the new protein to characterize its neighborhood, leading to a detailed map of the interactions of all *T*. *brucei* Nups, as well as to the discovery and characterization of five new Nups. Electron microscopy of Nups labeled with electron-dense gold provided further details on the locations of the Nups within the complex as a whole.

The most conserved portion of the *T*. *brucei* NPC was the inner ring, composed of a set of proteins that line the pore itself. The authors found that the overall architecture of the ring, and the positioning of individual Nups within it, matched that seen in vertebrates, yeast, and plants. Quite the opposite was true of the membrane-anchoring proteins:, no trypanosome orthologs were found of several Nups known from yeast and humans, and one of the new trypanosome Nups employed a transmembrane domain as part of its anchoring structure, which is not found in other eukaryotes.

Perhaps the most surprising discovery concerned the distribution of the FG-Nups, a set of proteins rich in phenylalanine (F) and glycine (G) residue repeats, which project into the central pore and are thought to interact with the proteins that mediate cargo transport. In vertebrates, yeast, and plants, a quarter of FG-Nups are restricted to either the cytoplasmic or nucleoplasmic faces of the NPC, and that asymmetry helps drive export of messenger RNA. In *T*. *brucei*, the FG-Nups were largely symmetrically distributed, including the ortholog of a cytoplasmically restricted protein in yeast, implying a different mechanism of mRNA export.

The confirmation that the structure of the inner core is highly conserved strengthens the model of NPC evolution previously proposed by the authors, in which the ancestral complex arose from multiple duplication events of a much simpler monomer that coated and stabilized openings in the nuclear membrane. The divergent membrane anchoring systems likely arose or were elaborated after the trypanosomes separated from the rest of the eukaryote pack, while the more symmetric distribution of their FG-Nups may reflect an ancestral condition, which the authors suggest was followed by development of asymmetry.

## Abbreviations

F: phenylalanine
G: glycine
GFP: green fluorescent protein
NPC: nuclear pore complex

## References

[1] Obado SO, Brillantes M, Uryu K, Zhang W, Ketaren NE, Chait BT, et al Interactome mapping reveals the evolutionary history of the nuclear pore complex. PLoS Biol. 2016;14(1): e1002365 doi: 10.1371/journal.pbio.1002365 26891179 10.1371/journal.pbio.1002365 PMC4758718

